# Postprandial effects of a polyphenolic grape extract (PGE) supplement on appetite and food intake: a randomised dose-comparison trial

**DOI:** 10.1186/s12937-015-0085-1

**Published:** 2015-09-14

**Authors:** Hyun-San Shin, Sophie Kindleysides, Wilson Yip, Stephanie C. Budgett, John R. Ingram, Sally D. Poppitt

**Affiliations:** 1 Human Nutrition Unit, School of Biological Sciences, University of Auckland, Auckland, New Zealand; 2 Department of Statistics, University of Auckland, Auckland, New Zealand; 3 Plant and Food Research Ltd, Mt Albert, Auckland, New Zealand; 4 Human Nutrition Unit, School of Biological Sciences, Department of Medicine, University of Auckland, Auckland, New Zealand

## Abstract

**Background:**

There is recent evidence that glucose delivered to the distal small intestine (SI) may stimulate the ileal brake and inhibit appetite. High polyphenolic grape extract (PGE) has been shown to inhibit α-amylase and α-glucosidase activity, two key enzymes required for starch digestion, *in vitro*. It is hypothesised to slow digestion and absorption of starch in the proximal SI such that glucose may be delivered distally into the ileum and suppress appetite. This study investigated the safety and efficacy of a PGE supplement, delivered within a capsule and consumed with a high-starch breakfast, on appetite ratings and *ad libitum* energy intake (EI) at a subsequent lunch meal.

**Methods:**

Twenty healthy, non-obese (BMI 18–28 kg/m^2^) male volunteers participated in a randomised, double blind, placebo controlled, three arm, cross-over study. Participants were administered (i) low dose PGE_500_ (500 mg), (ii) high dose PGE_1500_ (1500 mg), and (iii) matched placebo with a 2MJ high-starch breakfast (white bread); followed 3 h later by a single item buffet-style lunch meal (pasta and meat sauce). Outcome variables were feelings of hunger, fullness, prospective thoughts of food (TOF) and satisfaction assessed using visual analogue scales (VAS); and *ad lib* energy and macronutrient intake at the lunch meal.

**Results:**

There was no detectable effect of PGE_500_ or PGE_1500_ compared with placebo (all, time*supplement interaction, *P* > 0.05) on VAS-assessed hunger, fullness, TOF or satisfaction. There was also no evidence that PGE significantly altered *ad lib* energy or macronutrient intake at the lunch meal relative to placebo (*P* > 0.05). EI following PGE_500_ was +164 kJ higher than placebo (+5.3 %, *P* > 0.05); and EI following PGE_1500_was −51 kJ lower than placebo (−1.7 %, *P* > 0.05).

**Conclusions:**

Whilst well tolerated, there was no evidence that encapsulated low dose PGE_500_ or high dose PGE_1500_ consumed with a high starch breakfast meal altered postprandial hunger, fullness, TOF or satisfaction relative to a matched placebo. Nor was there evidence that either dose altered *ad lib* energy or macronutrient intake at an outcome meal.

**Trial registration:**

ACTRN12614000041651

## Background

Weight gain and obesity have emerged as a significant problem currently affecting global public health. The current therapeutic options for the overweight and obese are limited as most lack efficacy or are associated with adverse side-effects [1–3]. Satiety plays an important role in the maintenance of body weight and prevention of obesity as it allows the regulation of food intake on a meal to meal basis [4]. Amongst the numerous interacting mechanisms contributing to the regulation of satiety, enteroendocrine signals originating from the gastrointestinal (GI) tract are hypothesised to play an important role [5–7].

One hypothesis is focused on signals that may arise from the ileum, the most distal part of the small intestine (SI), or hind gut and there has been interest in dietary available CHO as a potential trigger for satiety signals arising from the ileum. There is a growing body of evidence from both animal and clinical studies that infusing glucose directly into the ileum can modify a range of GI activities, and that in turn this may put a brake on food intake [8]. However, achieving this effect with food is extremely challenging as the digestion and absorption of CHO occurs mainly in the stomach, duodenum and jejunum of the proximal SI, or fore gut, resulting in minimal delivery of monosaccharide glucose into the ileum [8–10]. The initial step in the enzymatic digestion of starch involves cleavage of the glucose-glucose α-(1, 4) glycosidic bond by α-amylase to produce shorter dextrin chains and glucose-glucose disaccharides (such as maltose and isomaltose). These products are then further hydrolysed into individual glucose units by a set of brush border enzymes collectively called α-glucosidases. Only these monosaccharide forms of CHO are then taken up by gut epithelial cells using specific transporters, sodium/glucose cotransporter 1 (sGLT1) and glucose transporter 2 (GLUT2) [11, 12].

Polyphenols are a structural class of plant-origin compounds characterized by the presence of large multiples of phenol structural units. The number and characteristics of these phenol structures underlie the unique physical, chemical, and biological (metabolic, toxic, therapeutic) properties of particular members of the class. The health benefits of polyphenols have been extensively studied and implicated as protective agents in a number of chronic diseases, including cardiovascular disease (CVD), diabetes and cancer [13–15]. Recently, a variety of polyphenols have been shown to exert biological effects in CHO metabolism by inhibiting the activity of α-amylase and α-glucosidase, two key enzymes required for starch digestion [14, 16–20]. On this basis, it is plausible that plants rich in polyphenols, when given with a high starch-based meal, may be able to induce CHO (glucose) malabsorption in the fore gut. This mechanism may allow transit of available CHO further down the GI tract, possibly as far as the hind gut, which in turn may result in the activation of satiety responses. A similar mechanism is utilized by the pharmaceutical industry in diabetic therapies such as acarbose or miglitol, which block CHO uptake in the fore gut to ameliorate glycaemia, and where in turn there is some evidence of appetite suppression [21, 22].

Grapes contain high concentrations of bioactive polyphenols [23]. Notably, recent *in vitro* studies have shown polyphenolic grape extracts (PGE), including grape seed extract (GSE), to be associated with significant inhibition of activity of α-amylase and α-glucosidase with the potential to prevent the breakdown of starch into its glucose components, blocking a step that is required for the absorption of dietary available CHO [23–25]. The objective of the present study was to test whether PGE, hypothesised to induce post-meal CHO malabsorption in the fore gut when consumed with a starch-rich food, would enhance satiety and reduce food intake at a later meal. Specifically, it aimed to compare the effects of low-dose (500 mg) and high-dose (1500 mg) PGE protected from gastric acid by delivery within a capsule and consumed with a standardised high-starch breakfast meal, on postprandial appetite and food intake.

## Methods

### Participants

Twenty healthy men (BMI 18–28 kg/m^2^) aged between 18 and 60 years were recruited in Auckland, New Zealand during January and February 2014, through poster and electronic advertisement. Participants were non-smokers, had no history of CVD, diabetes, or any other significant metabolic, endocrine or GI disease, and were not taking any medications that may have had any effect on appetite or weight regulation throughout the trial period. Other exclusion criteria included participation in an active diet program or loss/gain of >5 kg body weight within the last 6 months. Hypersensitivities or allergies to any foods or ingredients included in the study, as well as dislike and/or unwillingness to consume items listed as study foods (breakfast and lunch meals), unwilling/unable to comply with study protocol, or current participation in another clinical intervention trial were also exclusions. Participants were ascertained healthy by self-report during a screening visit. Human ethics consent was obtained from the New Zealand Health and Disability Ethics Committee (HDEC, Reference Number: 13/NTA/225). The trial was registered with the Australia New Zealand Clinical Trials Registry (ANZCTR), clinical trial registration number ACTRN12614000041651, and conducted at the Human Nutrition Unit (HNU), University of Auckland, New Zealand. Prior to registration for the trial, all participants provided written informed consent and were instructed that they were able to withdraw from the trial at any time.

### Study design, supplements, and protocol

This was a randomised, placebo controlled, double blind, three condition, cross-over study. Two doses of dried PGE (500 mg, 1500 mg) and a matched placebo (magnesium stearate, Mg(C_18_H_35_O_2_)_2_) were protected from gastric acid by encapsulation, using size ‘0’ capsules (Capsugel®, Morristown, USA, see Table 1), and administered as part of a standardised 2MJ high-starch, low-polyphenol breakfast (185 g white bread, Tip Top Super Soft®, containing 83 g polysaccharide starch). A commercial source of PGE was used in this trial (New Zealand Extracts Ltd, Marlborough, New Zealand) which was an extract from red and white grape seeds and skins extracted using a proprietary water-based method. Total polyphenolic content was measured using HPLC according to Association of Official Analytical Chemists (AOAC) 15^th^ Edition, 952.03 methods standard [26]. Each capsule contained 353 mg polyphenolics per 500 mg PGE, hence total polyphenol content was ~70 % by weight. The predominant phenolic components in the extract identified by HPLC were oligomeric proanthocyanidins (OPC, which were >40 % of total phenolics), epicatechin and epicatechin gallate. NZ Extracts Ltd also undertook blinding of the capsules, correct allocation of which was subsequently confirmed by the research team upon completion of the trial using capsules taken from the original source. The intervention arms were: standardised breakfast plus low dose PGE (PGE_500_: 1 PGE capsule plus 2 placebo capsules); standardised breakfast plus high dose PGE (PGE_1500_: 3 PGEcapsules); and standardised breakfast plus placebo (Placebo: 3 placebo capsules); hence 3 capsules were administered on each occasion to ensure the blind was maintained. The trial was not unblinded until completion of both the intervention and statistical analyses. Each study visit was separated by a washout period of at least 3 days where participants were free to resume their usual diet and exercise patterns.

**Table 1:** Composition of the polyphenolic grape extract (PGE)

	Total number of capsules administered	PGE content per capsule (mg)	Total PGE content (mg)	Total polyphenol content (mg)	Oliomeric proanthocyanidins (OPC) %
PGE_500_	1 PGE + 2 placebo	500	500	353	>40 %
PGE_1500_	3 PGE	500	1500	1059	>40 %
Placebo	3 placebo	0	0	0	0

The PGE supplement was a commercial extract from red and white grape seeds and skins, extracted using a proprietary water-based method and provided by New Zealand Extracts Ltd (Marlborough, New Zealand). The predominant phenolic component in the extract was oligomeric proanthocyanidin (OPC), comprising >40 % of total polyphenols. Total polyphenol content was measured using Association of Official Analytical Chemists (AOAC) 15^th^ Edition 952.03 standard methods. Placebo comprised magnesium stearate matched by capsule number, capsule weight and capsule size (size ‘0’) to the PGE treatments

The trial was conducted according to the standard methodologies of Blundell et al. [27] for the assessment of postprandial appetite response and subsequent eating behaviour, using a preload design. On the morning of each study day, participants arrived at the HNU at 0830 h after an overnight fast and baseline subjective appetite ratings were measured (*t* = 0). The standardised high starch breakfast was served at 0900 h with the test capsules and 250 mL of water. Participants were asked to consume the meal in full, but at their own pace, within 15 min. No further foods were allowed throughout the morning and the participants remained within the HNU until an *ad libitum* lunch was served at 1200 h (180 min later, *t* = 180). Appetite ratings were measured throughout the morning and for 2 h after completion of the lunch. The *ad lib* lunch was served in individual sensory booths, no distractions were allowed during the 30 min lunch period, and participants were instructed to eat until they felt comfortably full. Participants remained at the HNU throughout each study day and were allowed to read, use laptop computers or undertake other similar sedentary activities but were not allowed to sleep.

**Fig. 1:**
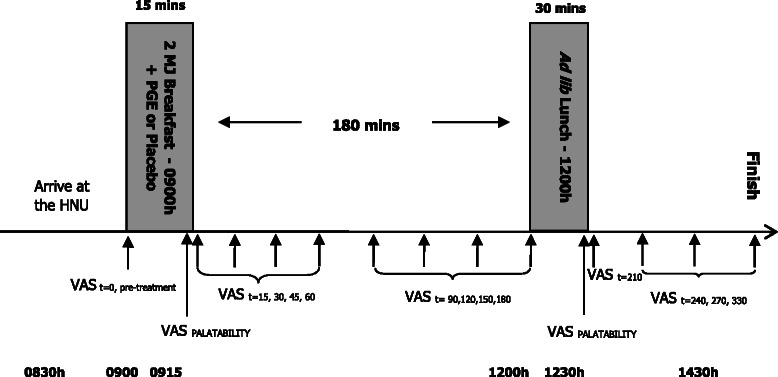
Daily protocol. Participants were given a standardised 2MJ high-starch breakfast plus PGE treatment or placebo capsules at 9am, then offered a lunch meal 3 hours later which they were encouraged to eat ad libitum until until they felt comfortably full. Visual analogue scores (VAS) were assessed throughout the day. Participants were restricted to the research clinic and allowed to consume only foods and beverages provided within the study. PGE, polyphenolic grape extractᅟ

For the measurement of energy and macronutrient intake, foods were weighed before and after the lunch meal. Throughout the day participants rated hunger, fullness and other appetite-related sensations, including satisfaction, current thoughts of food (TOF), thirst and nausea. Palatability of the breakfast and *ad lib* lunch was assessed following each respective meal. The daily protocol of the study is shown in Fig. 1.

### Appetite ratings

Subjective measures of hunger, fullness, satisfaction, TOF and nausea were the primary outcome in this trial, and were measured using validated visual analogue scales (VAS) according to the standard methodology of Blundell et al. [27] and previously described by our research group [28–30]. The trial was powered to detect a change in VAS of 10 % relative to placebo as statistically significant. The following questions were asked: “How hungry do you feel?/How full do you feel?/How satisfied do you feel?/How much do you think you can eat now/How nauseated do you feel?” anchored on the left by “I am not hungry at all/I am not full at all/I am completely empty/nothing at all/not nauseas at all” and “I am as hungry as I have ever been/I am totally full/I cannot eat another bite/a large amount/very nauseated” on the right. Participants marked their responses by placing a vertical line across the 100-mm scale according to their subjective feelings. Moreover, a set of scales rating how thirsty, energetic and relaxed the participants felt were included as a distraction from the main outcome. VAS were measured at 15, 30, 45, 60, 90, 120, 150, 180 (*ad lib* lunch), 210, 240, 270 and 330 min after the breakfast was served. Immediately after the breakfast and the *ad lib* lunch meal participants also rated the pleasantness, visual appeal, smell, taste, aftertaste and overall palatability on separate 100-mm VAS.

### Standardised breakfast and *ad libitum* lunch

The standardised breakfast comprised 185 g of sliced white bread, as shown in Table 2. The *ad lib* outcome meal was a lunch which comprised a restricted item savoury buffet consisting of beef and tomato sauce, boiled pasta spirals, and bottled water. EI at the outcome meal was a secondary outcome, with sample size adequate to detect a change of ~0.5MJ as statistically significant. In an attempt to avoid over-consumption the variety of meal items offered was limited, each item served in moderate excess with the intent that participants would not consume the entirety of either single item. We have previously demonstrated that this protocol is sensitive to changes made in a prior test preload [31]. Prior to the study, it had been established with each participant that the items provided in the *ad lib* lunch were acceptable as meal choices. Participants were advised that they could eat as much or as little as they chose and to eat until they were comfortably full. The lunch meal was served in individual booths to ensure that all participants remained undisturbed during the meal time, where they were required to remain for a period of 30 min. Lunch items were weighed both immediately before and after consumption of the meal. Energy, fat, CHO and protein intake were calculated using the dietary software program FoodWorks™ (Professional Edition, Version 5, 1998–2007, Xyris Software, Australia).

**Table 2:** Composition of the preload breakfast and *ad libitum* outcome lunch meal

	Weight (g)	Energy (kJ)	CHO (g)	CHO (%)	Fat (g)	Fat (%)	Protein (g)	Protein (%)
Breakfast								
White bread, sliced, no crusts	185	1943	^a^89	78	4	8	15	14
Water, bottled	250	0	0	0	0	0	0	0
*Ad lib* lunch								
Meat sauce, beef & tomato	1385	3858	55	25	39	38	83	37
Pasta, spirals, boiled	960	5662	278	82	6	4	45	13
Water, bottled	250	0	0	0	0	0	0	0

*CHO* carbohydrate; ^a^83g polysaccharide starch

### Statistical analyses

Data on demographic and anthropometric characteristics were summarized using descriptive statistics, and presented as mean, standard deviation (mean, SD). Power calculations confirmed a sample size of 20 individuals to be sufficient to detect a 10 % change in the primary outcome, VAS hunger/fullness, as statistically significant in this pair wise, cross-over design. Based on this sample size, a change in EI at the outcome lunch meal of ~0.5MJ or greater was expected to also reach significance. Efficacy endpoints of VAS and EI were presented as mean, standard error of the mean (mean, SEM). VAS data assessing feelings of hunger, fullness and other satiety indicators throughout the study days as well as VAS data assessing the palatability of the breakfast and lunch meals were analysed using repeated measures Linear Mixed Model ANOVA (SAS: PROC MIXED, SAS version 9.2, SAS Institute Inc, Cary, NC, USA, 2002–2008). The energy and macronutrient intake data from the outcome meal following each of the three supplement arms was also analysed using ANOVA. Intervention arm, participant and study day were included in the procedure, in addition to the supplement/time interaction which addressed whether the trajectory of VAS over time during the study period differed between the three arms (diet*time). Statistical significance was set at *P* < 0.05.

## Results

### Participants

Twenty male participants were screened, enrolled and randomised into this cross-over trial. The men were lean and healthy with a mean age of 26.4 years (1.7, SD) and a mean BMI of 23.1 kg/m^2^ (0.7, SD). All 20 participants completed the three study arms with no drop outs, exclusions or any lost to follow up. There were no reports of abdominal discomfort, nausea or other adverse GI symptoms during the study, indicating that both low (PGE_500_) and high (PGE_1500_) doses were well tolerated.

### VAS-rated appetite perceptions

**Fig. 2:**
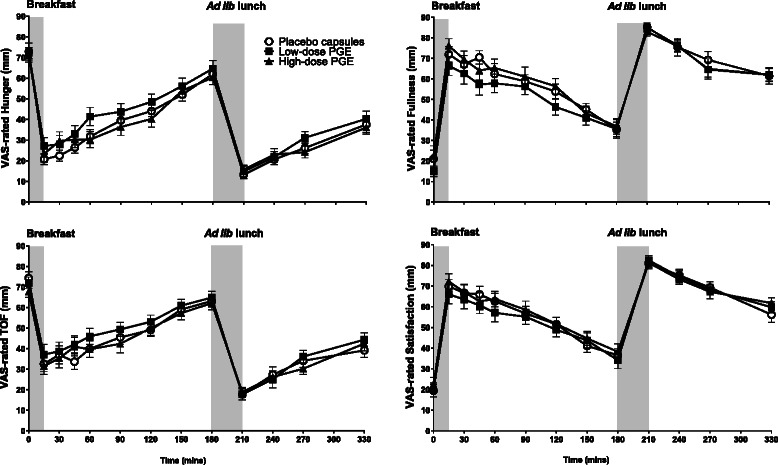
Mean (SEM) VAS-rated hunger, fullness, thoughts of food (TOF) and satisfaction throughout the day in response to 3 breakfast treatments: placebo; low-dose PGE (PGE500, 500 mg); high-dose PGE (PGE1500, 1500 mg). PGE, polyphenolic grape extractᅟ

The mean VAS-rated changes in hunger, fullness, TOF and satisfaction measured throughout each study day on each of the three arms are shown in Fig. 2. Fasting baseline scores for hunger, fullness, TOF and satisfaction did not differ between the three supplements, confirming that participants were in a comparable appetitive state at the start of each study day (all, *P* > 0.05). As expected, prior to consumption of the breakfast, scores for hunger were high and fullness low, a trend observed across all three arms. Consumption of the breakfast plus PGE or placebo capsules significantly decreased hunger and increased fullness scores on all 3 arms compared with baseline scores (all, *P* < 0.05). The VAS-rated changes in satisfaction and TOF were similar and consistent with those of hunger and fullness (Fig. 2, all, *P* < 0.05). The plots show consistent and predictable changes in line with known physiological effects. However, there was no significant difference between PGE supplements and inactive placebo in any of the VAS assessments either over the 180 min period between breakfast and lunch or throughout the full study day (time*supplement, all, *P* > 0.05), confirming that there was no effect of either PGE_500_ or PGE_1500_ on subjective measures of appetite compared with the inactive placebo.

### VAS-rated palatability and nausea

Participants rated the pleasantness, visual appeal, smell, taste, aftertaste and overall palatability immediately after the breakfast and the *ad lib* lunch meal (data not shown). In general palatability of the breakfast (white bread) was low, and palatability of the lunch (pasta and meat sauce) was high. There was no effect of any of the three intervention arms on these outcomes, hence no evidence of adverse sensory effects caused by administration of either the low- or high-dose PGE capsules when compared with inactive placebo. There was also no evidence of nausea induced by the PGE_500_ or PGE_1500_ capsules.

### Energy intake at *ad libitum* lunch

**Fig. 3:**
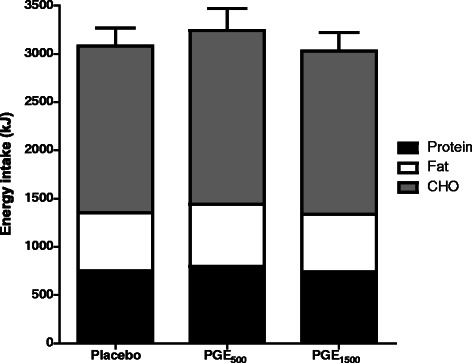
Mean (SEM) energy and macronutrient intake at the ad libitum lunch meal for each treatment PGE500, polyphenolic grape extract 500mg; PGE1500, polyphenolic grape extract 1500mg. CHO, carbohydrateᅟ

*Ad lib* EI at the lunch meal is presented for each study arm in Fig. 3. Mean (SEM) EI at lunch was 3079 (187) kJ, 3243 (225) kJ and 3028 (194) kJ for placebo, PGE_500_ and PGE_1500_, respectively. EI following PGE_500_ was +164 kJ higher than placebo (+5.3 %); and EI following PGE_1500_was −51 kJ lower than placebo (−1.7 %). There was no significant difference between supplements (ANOVA, *P* > 0.05), hence no evidence that PGE at either dose when delivered within a capsule and with a starch-rich breakfast meal significantly altered EI relative to placebo.

## Discussion

In this study we were interested in determining the effect that encapsulated PGE, proposed as a rich source of polyphenols, may have on the ileal brake mechanism of appetite control. Evidence from naso-ileal tube studies that delivery of nutrients directly to the distal SI may impose a brake on appetite has generated considerable interest [7, 8, 32], and lead to speculation that it may be possible to induce the ileal brake following a meal. The hypothesis that orally delivered monosaccharide glucose may avoid absorption in the duodenum and hence transit through to the distal SI and thereby enhance satiety was originally proposed following studies where pharmaceutical α-glucosidases were administered with a CHO-rich meal [21, 22]. Prescribed as oral therapeutics to promote better regulation of blood glucose and prevent significant postprandial glucose excursions in patients with impaired glucose tolerance (IGT) or type 2 diabetes (T2D), the α-glucosidases or’ starch blockers’ act to decrease the rate of starch digestion through reversible inhibition of pancreatic α-amylase and membrane-bound intestinal α-glucosidase [12, 33–35]. They include acarbose, voglibose and miglitol which essentially induce malabsorption by preventing or slowing the breakdown of complex CHO to glucose in the proximal SI, moving the absorption site of CHO to the distal portion of the intestine. In clinical studies where these starch blockers have been given with a high CHO-meal, the rate of gastric emptying has decreased and sustained enhancement of peptides associated with satiety has been reported, including glucagon-like peptide-1 (GLP-1) which is secreted predominantly from the ileal epithelial endocrine L-cells [21, 22, 36, 37]. These trials have also demonstrated suppression of appetite, albeit with mild to moderate GI adverse events [21, 22], attributed to malabsorption of CHO within the proximal SI and increasing concentration of glucose within the distal SI including the ileum. Therefore, it is conceivable that dietary sources of naturally occurring α-glucosidase and α-amylase inhibitors, such as those of plant-origin including grapes, may also suppress proximal SI glucose digestion allowing delivery of glucose distally.

PGE, amongst a number of other naturally occurring plant extracts rich in bioactive polyphenols, has been demonstrated *in vitro* to be a potent inhibitor of the α-glucosidases [24, 38, 39], but whether it could modulate appetite and EI had not previously been investigated. Interestingly, there are some prior animal studies which have reported a link between PGE and decreased food intake and body weight, although with no direct evidence that the effects were driven by change in appetite regulation. In an early study in rats, 12-week supplementation of grape seed extract (GSE) decreased food intake and prevented weight gain [40]. Notably, the authors attributed the results in part to a binding affinity of the polyphenols for specific proteins within the ingested food, forming aggregates in the GI tract and leading to delayed digestibility and absorption of the nutrients. In a more recent study rodents with obesity induced by a high fat diet were supplemented with GSE over 5 weeks, and reported prevention of further weight gain and improvement in obesity-related serum biochemistry such as circulating lipid profile [41]. Neither study attributed the effects on body weight to changes in appetite regulation, despite the changes in food intake. Our study was the first to assess the efficacy of PGE delivered within a capsule at the same time as a high starch breakfast meal and found that whilst PGE at doses of 500 and 1500 mg was safe and well tolerated, it did not significantly modulate appetite or EI when compared to a matched placebo.

There are several important methodological issues to be noted from our study. Grape extracts are widely consumed as a nutritional supplement worldwide, are generally regarded as safe (GRAS) [14, 15, 42] and in our present study we were able to confirm the tolerability of PGE doses ranging from 500 to 1500 mg in healthy males. Notably, suppression of food intake was observed in the rodent trials at a dose of between 70 and 100 mg/kg GSE [40, 41]. Body surface area (BSA) normalisation algorithms [43], albeit a simple approach to dose conversion [44], show this to be to a human equivalent dose (HED) of ~350–1,000 mg in a 60 kg adult (HED, mg/kg = rodent dose, mg/kg × [rodent K_m_/human K_m_], where mouse K_m_ = 3, rat K_m_ = 6, human K_m_ = 37), and indicate that sufficient dose may have been administered in our current trial. Secondly, a lack of information on the absorption and bioavailability of PGE-derived polyphenols in humans raised a challenge in our current study, where the intent was to orally deliver sufficient concentration of polyphenols into the proximal SI in order to delay CHO absorption. We did not assess this outcome in this trial. Thirdly, partially blocking starch breakdown may not have been sufficient to enable distal transit of dietary glucose which may also require inhibition of glucose uptake as it passed through the proximal SI.

## Conclusions

In our present clinical study, whilst we were able to confirm the tolerability of PGE doses ranging from 500 to 1500 mg in a group of healthy males, there was no evidence that supplementation with PGE altered postprandial appetite response to a starch-rich breakfast meal nor *ad lib* energy or macronutrient intake at a later lunch meal, relative to a matched placebo. It is not known whether PGE can delay digestion and absorption of available CHO and enhance delivery of glucose to the ileum in a clinical setting. Whether PGE administered at different time intervals (pre or post CHO meal), at higher or repeated doses over a longer time period, or synergistically with glucose uptake blockers, can alter satiety is not known and may be of interest for investigation in future trials.

## Abbreviations

ANOVA: Analysis of variance
BMI: Body mass index
BSA: Body surface area
CHO: Carbohydrate
CVD: Cardiovascular disease
EI: Energy intake
GI: Gastrointestinal
GLP-1: Glucagon like peptide-1
GLUT2: Glucose transporter 2
GSE: Grape seed extract
HDEC: Health and Disability Ethics Committee
HNU: Human nutrition unit
IGT: Impaired glucose tolerance
PGE: Polyphenolic grape extract
SD: Standard deviation
SEM: Standard error of the mean
SI: Small intestine
sGLT1: Sodium/glucose cotransporter 1
T2D: Type 2 diabetes
TOF: Thoughts of food
VAS: Visual analogue scale
